# Survey of electrically evoked responses in the retina - stimulus preferences and oscillation among neurons

**DOI:** 10.1038/s41598-017-14357-1

**Published:** 2017-10-23

**Authors:** David Tsai, John W. Morley, Gregg J. Suaning, Nigel H. Lovell

**Affiliations:** 1 Department of Electrical Engineering, Columbia University, New Yok, NY USA; 2 School of Medicine, Western Sydney University, Sydney, NSW Australia; 3 School of Medicine, UNSW Australia, NSW Australia; 4 Graduate School of Biomedical Engineering, UNSW Australia, NSW Australia

## Abstract

Electrical stimulation is an important tool in neuroscience research and clinically. In the retina, extensive work has revealed how the retinal ganglion cells respond to extracellular electrical stimulation. But little is known about the responses of other neuronal types, and more generally, how the network responds to stimulation. We conducted a survey of electrically evoked responses, over a range of pulse amplitudes and pulse widths, for 21 cell types spanning the inner two layers of the rabbit retina. It revealed: (i) the evoked responses of some neurons were charge insensitive; (ii) pulse-width sensitivity varied between cell types, allowing preferential recruitment of cell types; and (iii) 10–20 Hz damped oscillations across retinal layers. These oscillations were generated by reciprocal excitatory / inhibitory synapses, at locations as early as the cone-horizontal-cell synapses. These results illustrate at cellular resolution how a network responds to extracellular stimulation, and could inform the development of bioelectronic implants for treating blindness.

## Introduction

Electrical stimulation has a long application history in neuroscience research, for inferring the function of neurons individually and across brain areas^1,2^. More recently, it has been applied to treat a range of disorders in the central nervous system, ranging from implantable stimulators for neurodegenerative diseases^3,4^, deep brain stimulators for neurologic^5^ and neuropsychiatric disorders^6^, and brain machine interfaces^7^. In particular, the last two decades have witnessed rapid progress in the design and development of retinal implants for restoring sight to the profoundly blind^8–13^.

With sufficient strength, electrical stimulation activates neurons directly^14^. Because neurons are interconnected, the spatiotemporal consequences of electrical stimulation may extend far beyond the region immediately adjacent to the electrodes, and span a time scale significantly longer than the stimulus duration. Experimental and theoretical analyses^14–20^ have made significant contributions to our understanding of the biophysics behind electrical stimulation at the level of individual neurons in the retina. It has proven difficult, however, to formulate a systemic understanding on how large neural networks, such as the retina, respond to electrical stimulation with single-cell resolution. This is due primarily to the absence of a comprehensive survey on evoked responses for all neuronal types within the target network, across a range of stimulus configurations.

With the exception of three reports^21–23^, only the retinal ganglion cells (RGCs; the retina’s output neurons) have been recorded directly during retinal electrical stimulation studies. Other neuronal types, such as the bipolar cells, amacrine cells and horizontal cells, are expected to respond to electrical stimulation. Many of these neurons also survive in large numbers following neurodegenerative diseases^24,25^. However, because of challenging experimental access, there is a paucity of information on how these neurons in the inner retina respond to artificial electrical stimuli. Their electrically-evoked responses have largely been inferred through RGC post-synaptic currents or from RGC spikes. The handful of studies that directly recorded from these neurons have relied on slicing the retina^21,22^ or delaminating the photoreceptor layer^23^. This compromises network connectivity and involves stimulating-electrode-to-tissue placements that do not correspond to clinical arrangements. Finally, these studies either examined only the bipolar cells or did not identify the cell type.

Here we combined intracellular electrophysiology and morphological characterization to compile a survey of electrically evoked responses, for 21 neuronal types spanning the inner two retinal layers, and over a range of stimulus configurations. Next, analyses of this data revealed that: (i) the response amplitude of two wide-field neurons and horizontal cells did not scale with stimulus charge; (ii) sensitivity to pulse width differed between neuronal types, offering the possibility for preferential recruitment; and (iii) 10–20 Hz damped oscillations occurred across retinal layers following electrical stimulation. Finally, pharmacological manipulations and computational simulations revealed a simple connectomic substrate responsible for the oscillation – reciprocal excitatory / inhibitory synapses. The ubiquity of such connectivity implies that similarly damped oscillatory responses may occur following electrical stimulation in other parts of the central nervous system.

## Results

### A library of electrically evoked responses

**Figure 1:**
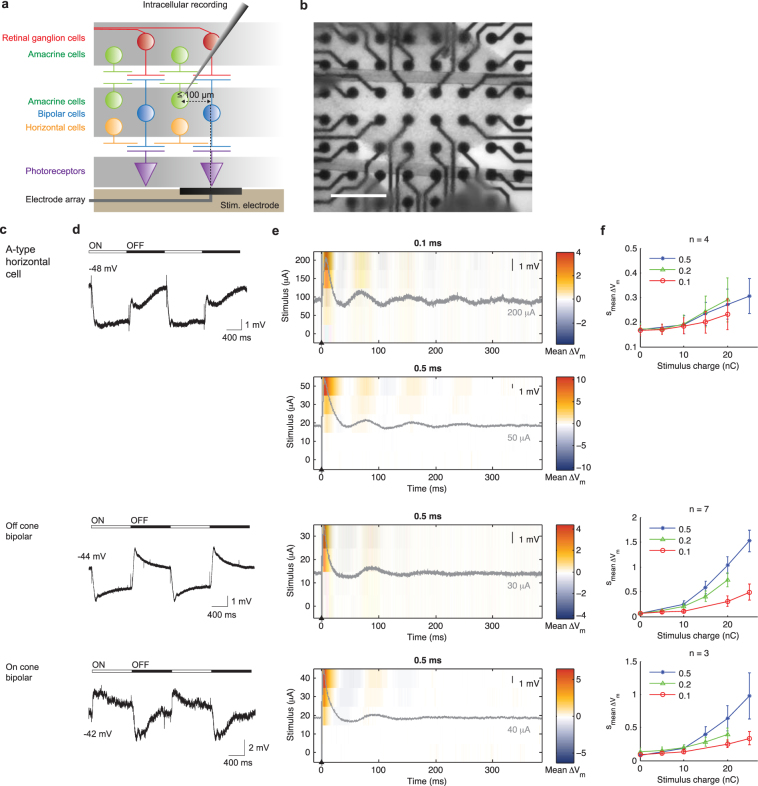
Light- and electrically-evoked responses of horizontal cells and cone bipolar cells. (**a**) We stimulated wholemount retinas at the photoreceptor-side (subretinal stimulation). Intracellular recordings were made for cell types throughout the retina, including: horizontal cells, bipolar cells, amacrine cells and retinal ganglion cells. (**b**) Photo of retina over the stimulation array. (**c**) Name of cell type and, when available, equivalent name in literature. (**d**) Representative response to 160 µm diameter flashing light spot. Resting membrane potential is also indicated. (**e**) Stimulus-time plot of electrically evoked responses, over time (horizontal axis), as a function of stimulus amplitude (vertical axis). The colors represent V_m_ variations. All color plot results are averaged over 10 trials. The plot title indicates the stimulus pulse width used. The overlaid grey traces are representative single-trial response at the strongest stimulus amplitude. (**f**) Average V_m_ perturbation (s_mean ∆Vm_) over all trials, for multiple cells, as a function of pulse width and pulse amplitude (stimulus charge).

We assembled a library of morphology, light evoked responses and electrically evoked responses for 21 cell types across the inner two layers of the rabbit retina, encompassing all major interneuron types, including horizontal cells, bipolar cells, amacrine cells, as well as the retinal ganglion cells (RGCs). The isolated rabbit retinas were placed photoreceptor-side down (Fig. 1a) on a multielectrode array (MEA) (Fig. 1b). Each neuron was characterized by intracellular recording and by morphology (Supplementary Figs 1–4). Light responses were evoked by flashing a spot over the neuron.

For extracellular electrical stimulation, one of the MEA electrodes was chosen as the stimulating electrode and eight distant electrodes around the MEA perimeter served as the stimulus return. For consistency, we only recorded from neurons ≤ 100 µm from the stimulation electrode center. For each neuron, we tested 0.1, 0.2 and 0.5 ms charge-balanced, cathodic-first, biphasic pulses, over a range of stimulus amplitudes. This permitted us to examine how the evoked responses varied as a function of pulse width and pulse amplitude.

### Horizontal cells and cone bipolar cells

**Figure 2:**
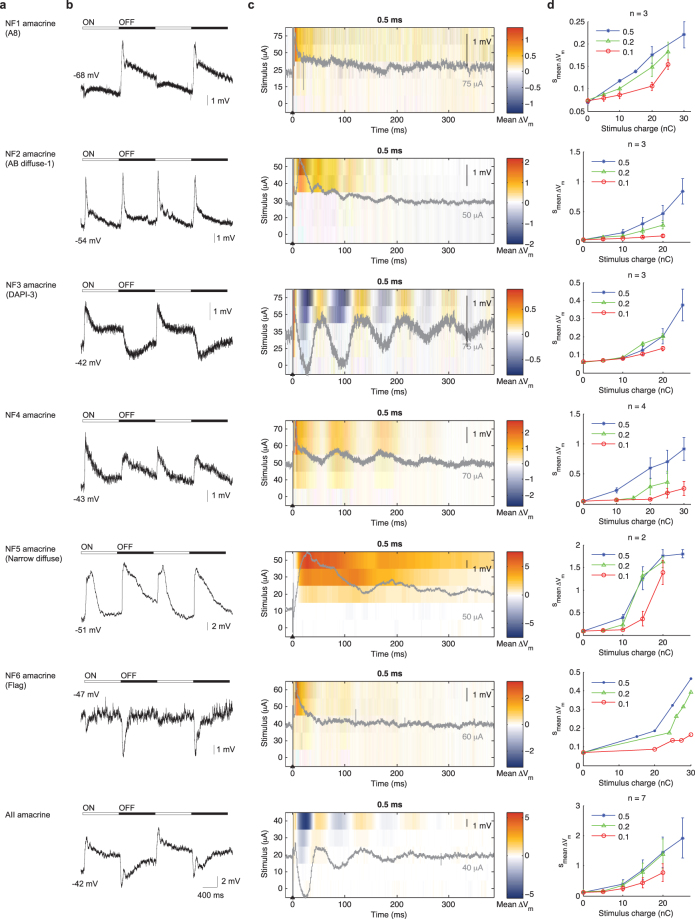
Light- and electrically-evoked responses of narrow field amacrine cells (dendritic arbor <125 µm). (**a**) Cell type name. (**b**) Light evoked responses. (**c**) Stimulus-time plots of electrically evoked responses. (**d**) Average V_m_ perturbation as a function of pulse width and pulse amplitude. See Fig. 1 legend for further details.

**Figure 3:**
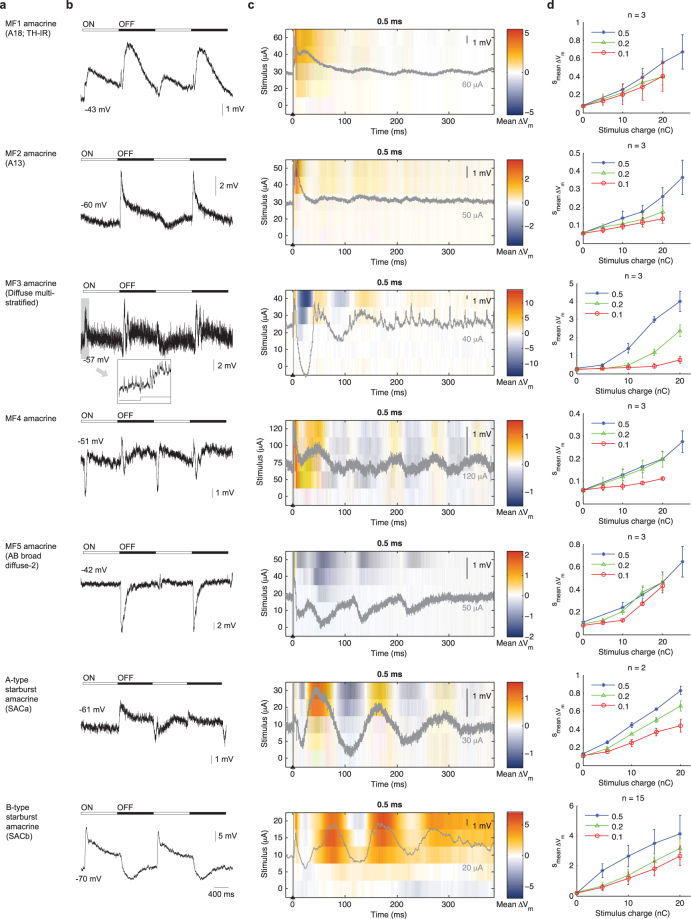
Light- and electrically-evoked responses of medium field amacrine cells (dendritic arbor 125–400 µm). (**a**) Cell type name. (**b**) Light evoked responses. (**c**) Stimulus-time plot of electrically evoked responses. (**d**) Average V_m_ perturbation as a function of pulse width and pulse amplitude. See Fig. 1 legend for further details.

**Figure 4:**
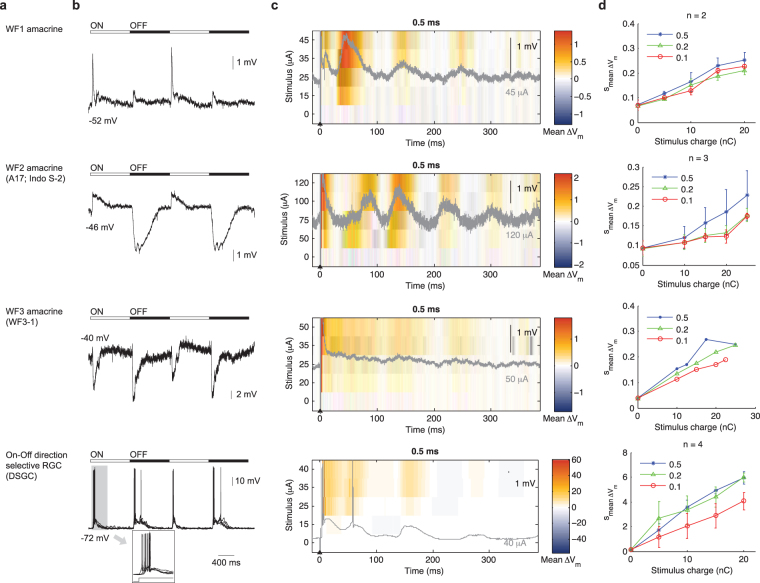
Light- and electrically-evoked responses of wide field amacrine cells (dendritic arbor >400 µm) and direction selective ganglion cells (DSGC). (**a**) Cell type name. (**b**) Light evoked responses. (**c**) Stimulus-time plot of electrically evoked responses. (**d**) Average V_m_ perturbation as a function of pulse width and pulse amplitude. See Fig. 1 legend for further details.

We began with the A-type horizontal cells (Fig. 1c) in the distal retina. Consistent with previous findings^26,27^, these neurons have a depolarized resting potential in darkness (−48 mV in Fig. 1d), and exhibited sustained hyperpolarization in response to the stationary light spot (Fig. 1d). Following a single extracellular stimulus, horizontal cells exhibited an initial transient depolarization of ~20 ms duration, followed by a series of damped oscillations, which decayed over several hundred milliseconds. This is illustrated in Fig. 1e as overlaid grey traces, for 0.1 ms and 0.5 ms biphasic pulses. We note that these long-duration, damped oscillations are of biological origin, rather than electronic artifacts, because they could be suppressed by pharmacological blockers (see section “Damped oscillations arose through synaptic interactions”) and their temporal kinetics varied between cell types (see also Figs 2c, 3c and 4c). To illustrate how the membrane potential varied over time, following stimulation, as a function of stimulus amplitude, we calculated the average deviation from resting potential over ten trials (${\rm{mean}}\,{\rm{\Delta }}{V}_{m}$) and plotted the data as color charts. The size of the initial transient response and the subsequent damped oscillation increased with stronger stimulus strength (Fig. 1e). These observations were consistent across all horizontal cells. This is summarized for all recorded horizontal cells in Fig. 1f, where we computed the standard deviation of the change in membrane potential, averaged over all recorded neurons, in the 390 ms analysis period (${{\rm{s}}}_{\text{mean}{\rm{\Delta }}{V}_{m}}$).

Next we examined the cone bipolar cells (Fig. 1c). The Off and On cone bipolar cells hyperpolarized and depolarized, respectively, in response to the light spot (Fig. 1d). Following extracellular stimulation, these neurons also responded with an initial transient depolarization, followed by damped oscillation (Fig. 1e), akin to the horizontal cells. However, given the same stimulus strength, cone bipolar cells’ responses were considerably larger than those of horizontal cells (Fig. 1f).

### Amacrine cells

The amacrine cells are the most diverse class of retinal neurons. We organized these neurons into three categories according to their dendritic field size^28^: narrow (<125 µm), medium (125–400 µm) and wide (>400 µm). Whenever possible, we matched our recorded neurons to documented amacrine cell types in the rabbit retina^28–34^ based on morphology and, if reported previously, also by light responses. The previously given names are shown between parentheses in Figs 2a, 3a and 4a.

The light-evoked responses (Fig. 2b) and electrically evoked responses (Fig. 2c) of narrow-field amacrine cells were extremely diverse. Similar diversity also held for the medium-field (Fig. 3) and wide-field (Fig. 4) amacrine cells. Nevertheless, several general observations could be made. First, a single extracellular stimulus elicited responses of several hundred milliseconds in most amacrine cells. Second, increasing the stimulus amplitude gradually increased the size of the evoked response. Third, electrical stimulation evoked oscillatory membrane potentials in most amacrine cells.

### Retinal ganglion cells

RGCs have been the primary focus of electrical stimulation studies to date^18–20,35–38^. To provide a reference for comparison against the existing studies, we examined the On-Off direction selective RGCs. These neurons respond to both the onset and the offset of light stimuli with spikes^39^ (Fig. 4b). Extracellular stimulation pulses of sufficient amplitude elicited a short latency spike (Fig. 4c), through direct activation of these neurons^18^. Subthreshold responses were observed across a range of stimulus amplitudes. Long latency spikes were often seen above the subthreshold responses (Fig. 4c). Increasing the stimulus charge increased the overall response magnitude over the 390-ms analysis time window (Fig. 4d).

### Wide-field neurons & horizontal cells are charge insensitive

**Figure 5:**
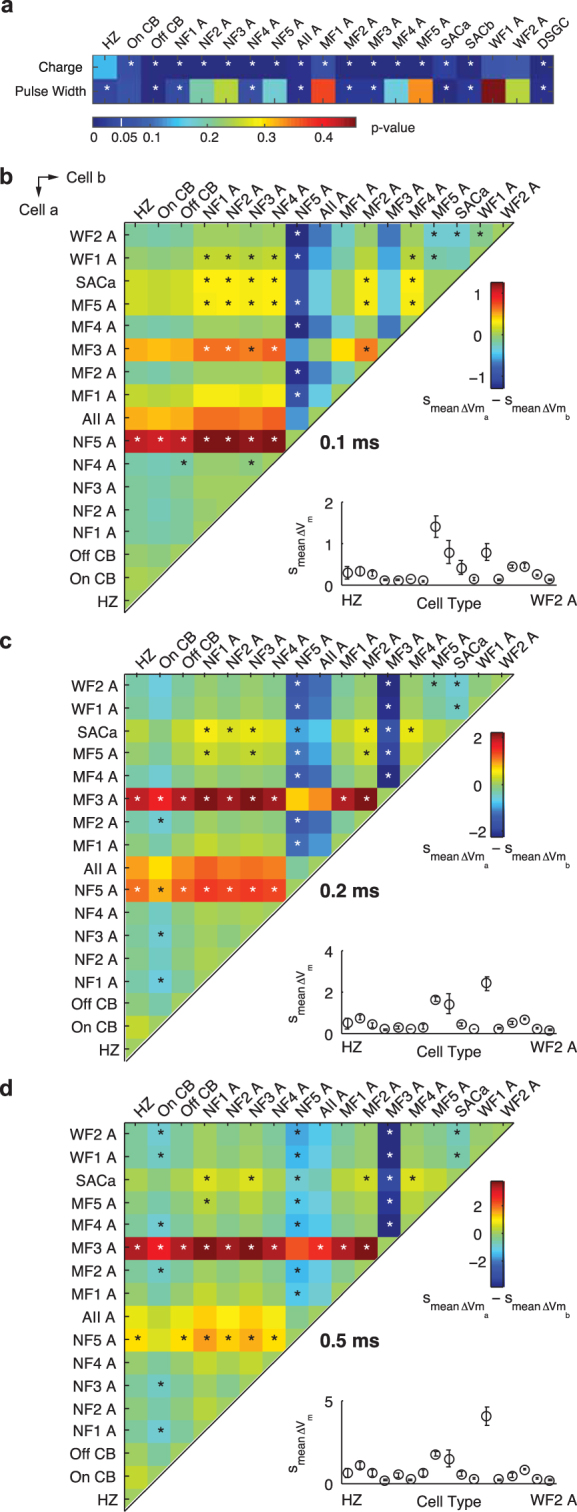
Within and between cell type comparisons of stimulus configurations. HZ, CB and A denote horizontal cell, cone bipolar cell and amacrine cells, respectively. The stars denote statistical significance (p < 0.05). (**a**) Increasing the stimulus charge elicited significantly larger response (s_mean ∆Vm_) for most cell types. The effect of pulse width (0.1–0.5 ms, with charge fixed at 20 nC) on response amplitude is only statistically distinguishable in some cell types (repeated measure 2-way ANOVA). (**b**–**d**) We pair-wise compared the response amplitude of every cell type against every other cell type (s_mean ∆Va_ − s_mean ∆Vb_). In each colored comparison plot the vertical and horizontal axis represents cell type ‘a’ and cell type ‘b’, respectively. At each pulse width, some neurons were more strongly driven (2-sample t-test) than the population. Insets: the response amplitudes (s_mean ∆V_) for each cell type.

The library we have assembled offers unique opportunities for within and between cell-type analyses. We asked if increasing the stimulus charge consistently resulted in larger responses, that is, larger membrane potential variations (${{\rm{s}}}_{\text{mean}{\rm{\Delta }}{V}_{m}}$) following pulse delivery. For each type of neuron, we compared the ${{\rm{s}}}_{\text{mean}{\rm{\Delta }}{V}_{m}}$ as the charge increased (repeated measure 2-way ANOVA, Fig. 5a). All but the horizontal cells and two wide-field amacrine cells exhibited significant increases in response amplitude with increasing charge (p < 0.05, marked by stars in Fig. 5a). Morphologically, these wide-field amacrine cells have dendrites spanning several hundred µm or more across the retina. The A-type horizontal cells also have broad-coverage neurites, and are connected to neighboring horizontal cells of the same type via gap junctions, forming a syncytium^40^. These neurons’ response amplitude (${{\rm{s}}}_{\text{mean}{\rm{\Delta }}{V}_{m}}$) increased by no more than approximately 2-fold despite the 20-times increase in stimulus charge.

### Some cell types are pulse-width sensitive

We next asked if stimulus pulse width had significant effects on the neurons’ response amplitude (${{\rm{s}}}_{\text{mean}{\rm{\Delta }}{V}_{m}}$). With the charge fixed at 20 nC for each cell type, we compared the ${{\rm{s}}}_{\text{mean}{\rm{\Delta }}{V}_{m}}$ as a function of pulse width (repeated measure 2-way ANOVA, Fig. 5a). Approximately half of the cell types showed significant differences in response amplitude between pulse widths (p < 0.05, marked by stars in Fig. 5a).

### Changing the most strongly driven cell type with pulse width

The prevalence of pulse width sensitivity among retinal neurons (Fig. 5a, charge fixed at 20 nC) raises the question of whether pulse width could be used to preferentially recruit neuronal types. We performed pairwise comparisons of electrically evoked membrane potential variations (${{\rm{s}}}_{\text{mean}{\rm{\Delta }}{V}_{m}}$) for each cell type against every other cell type, for 0.1–0.5 ms pulse width, while fixing the charge at 20 nC (Fig. 5b–d). Given the same stimulus, some cell types responded significantly more strongly than the rest of the population (2-sample t-test, *denotes p < 0.05 in Fig. 5b–d).

Using 0.1 ms pulses (Fig. 5b), the NF5 amacrine cells responded most strongly among the neuronal types. The response magnitudes of MF3, MF5, SACa (A-type starburst amacrine cell) and WF1 were also statistically higher than the rest of the population. As the pulse width was increased to 0.2 ms (Fig. 5c), then to 0.5 ms (Fig. 5d), the most responsive cell type shifted from NF5 to MF3. Concomitantly, the responses of MF5, SACa and WF1 amacrine cells decreased with increasing pulse width. Thus, pulse width variations changed the most strongly driven cell types, offering opportunities for preferentially activating certain cell types.

### Electrical stimulation evokes damped oscillation

**Figure 6:**
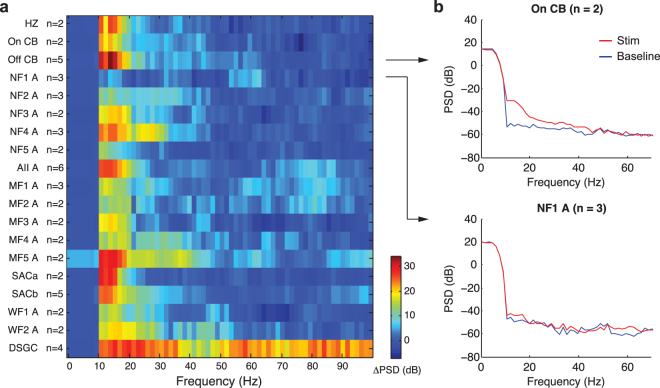
Extracellular stimulation elicited damped oscillations across retinal layers. The stimuli were 20 nC and 0.5 ms in duration. PR, HZ, CB, A and RGC denotes photoreceptor, horizontal cell, cone bipolar cell, amacrine cell and retinal ganglion cell. (**a**) Power spectrum of electrically evoked responses. This was computed by subtracting the spectrum during quiescence (b, blue trace) from the spectrum with electrical stimulation (b, red trace). (**b**) The change in power spectral density was clear in On cone bipolar, while minimal in NF1 amacrine.

A brief extracellular stimulus elicited dramatic, damped oscillations in the majority of cell types we examined, lasting several hundred milliseconds (Figs 1e, 2c and 4c). To precisely quantify these oscillations, we computed the difference in response power spectral density (PSD) with and without electrical stimulation (PSD_stim_ − PSD_baseline_). We observed an increase in power over the 10~20 Hz range for most neuronal types (Fig. 6a). Notably, the damped oscillations were widespread throughout the network. As a comparison to rare cell types with little oscillation, the change in PSD of NF1 amacrines was minimal following electrical stimulation (Fig. 6b).

### Damped oscillations arose through synaptic interactions

**Figure 7:**
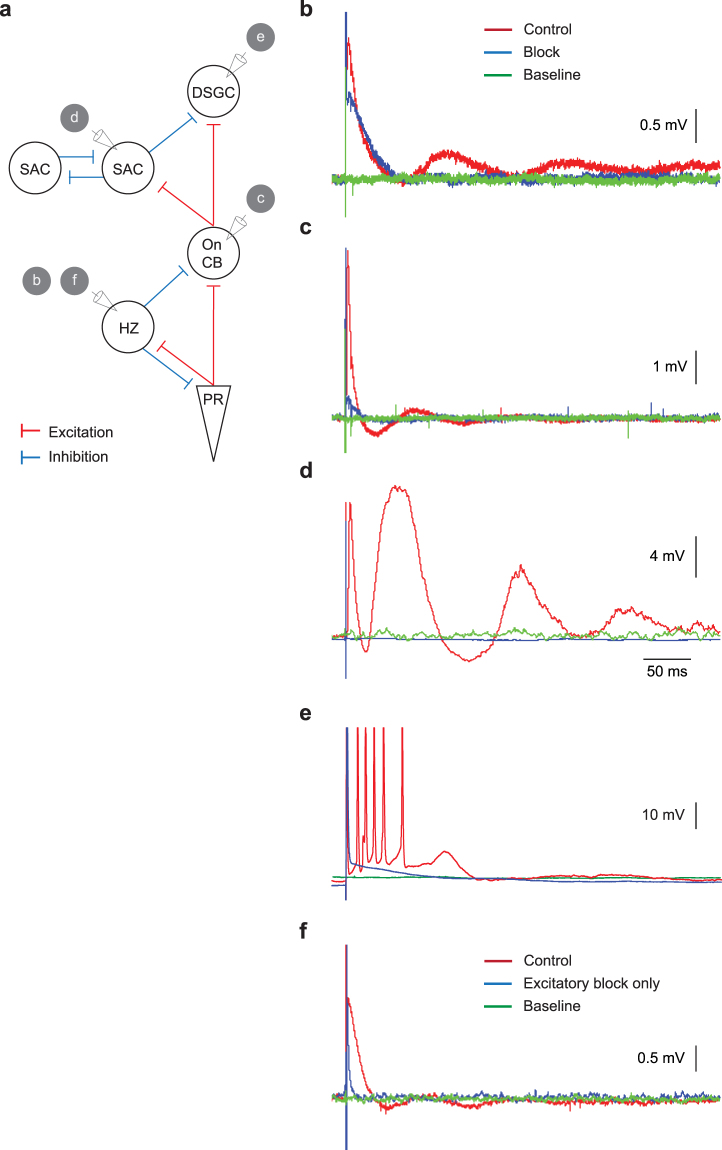
Damped oscillation arose through synaptic interactions. (**a**) To explore the mechanisms and location of damped oscillations, we focused on the direction encoding pathway. It contains: cone photoreceptors (PR), A-type horizontal cells (HZ), On cone bipolar cells (On CB), B-type starburst amacrine cells (SAC) and On-Off direction selective ganglion cells (DSGC). Representative extracellular stimulation responses under standard condition (red), during excitatory and inhibitory synaptic blocking (blue, with CNQX + MK-801 + L-AP4 + PTX + STY) and during quiescence without stimulus (green) for: horizontal cells (**b**), On cone bipolar cells (**c**), B-type starburst amacrine (**d**) and DSGC (**e**). (**f**) Representative horizontal cell responses during standard stimulation condition, blocking of excitatory synapses and quiescence.

How did the widespread, damped oscillation arise? Oscillations could be generated by: (i) spontaneously oscillating neurons^41^; (ii) neurons that resonate in response to a stimulus through ionic channel mechanisms^42^; and/or (iii) network interaction of neurons, which in isolation are incapable of oscillation^43^. A thorough analysis on the origin(s) of the damped oscillation is hampered by the lack of network wiring information for the majority of amacrine cell types. To address this question, we therefore focused on the direction encoding pathway in the retina, where the connectivity has been explored extensively^39^. This pathway (Fig. 7a) consists of photoreceptors, horizontal cells, cone bipolar cells, starburst amacrine cells and direction selective RGCs.

None of these neurons are known to oscillate spontaneously. Consistent with this notion, baseline recordings without stimulus revealed stable membrane potentials (green traces in Fig. 7b–e). Therefore, mechanism-(i) cannot be responsible for the damped oscillation.

We next recorded from the same neurons with Ames’ Medium containing glutamatergic, GABAa/c and glycinergic blockers. The neurons lost the ability to oscillate when they were synaptically isolated. Besides the displaced starburst amacrine cell, all neurons also continued to exhibit an initial depolarizing transient in response to the extracellular stimulus. These observations ruled out mechanism-(ii) – intrinsic ionic channel properties. Importantly, these results also suggest that synapses – mechanism-(iii) – are crucial for the damped oscillations.

### Cone-horizontal-cell reciprocal synapses generate damped oscillation

Knowing the putative mechanism responsible for the damped oscillation, we next searched for the locations within the direction encoding network where it first arises. Systematic recordings across the retina (Fig. 7a) indicated that the damped oscillation was already present at the horizontal cells (red traces, Fig. 7b). Horizontal cells receive excitatory inputs from the photoreceptors and provide inhibitory feedback onto the photoreceptors. Disrupting this reciprocal connectivity with glutamatergic blockers alone was sufficient to eliminate the damped oscillation (Fig. 7f), further supporting the notion that synaptic interactions underlie the oscillatory responses.

### Horizontal cell syncytium modulates damped oscillation

**Figure 8:**
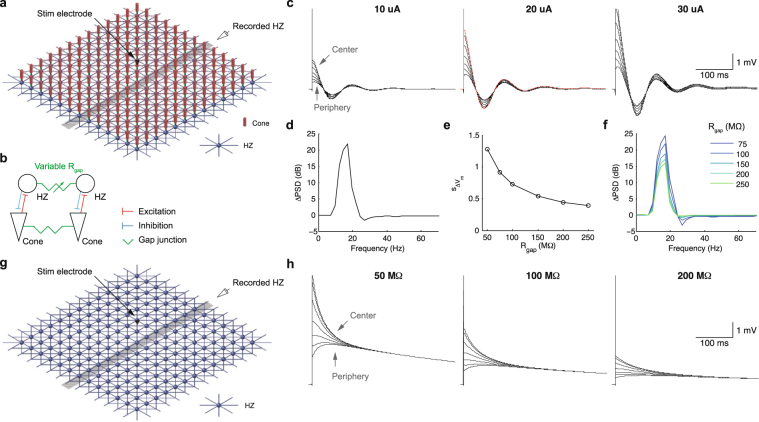
Computational simulation of damped oscillation in the photoreceptor and horizontal cell layer. (**a**) Computational network model containing cones and horizontal cells (HZ). The electrical stimuli were placed above the network center (black cone). The responses for a row of horizontal cells were monitored (highlighted in gray). (**b**) Architecture of the synaptic connectivity and gap junctions. (**c**) HZ responses to 0.5 ms stimuli of various current amplitudes. (**d**) Response power spectrum of the center HZ (red in c). (**e**) Center HZ V_m_ perturbation (s_∆Vm_) by a pulse (20 µA, 0.5 ms), as a function of HZ gap junctional resistance, R_gap_. (**f**) Response power spectra of the center HZ as a function of R_gap_. (**g**) Network model containing only HZ and gap junctions. (**h**) No oscillatory response was observed in a HZ-only network, over a range of junctional resistances. The stimuli were 0.5 ms pulses of 20 µA amplitude.

Cones and horizontal cells are interconnected to adjacent cones and horizontal cells, respectively, with gap junctions. In particular, the horizontal cell gap junctional conductance is modulated by exogenous factors^40,44^. What roles does this conductance play in the electrically evoked responses of horizontal cells? To address this question, we constructed a computational model consisting of the cone and horizontal cell layer (Fig. 8a). Both layers were internally connected by gap junctions (Fig. 8b). The cones and horizontal cells were connected by excitatory and inhibitory reciprocal synapses. We then placed an extracellular stimulation electrode above the photoreceptor layer, over the center of the grid, and observed the activities of a row of horizontal cells.

Following an extracellular stimulation pulse, the horizontal cells responded with damped oscillation (Fig. 8c) spanning the 10–20 Hz range (Fig. 8d), similar to those of intracellular recordings (Fig. 7b). Cells in the grid center, directly beneath the stimulation electrode, had larger evoked responses than those at the periphery. Increasing the stimulus increased the horizontal cells’ response amplitude, again consistent with intracellular recordings (Fig. 1e).

The oscillation amplitude of horizontal cells decreased with increasing gap junctional resistance (Fig. 8e), approaching an asymptotic limit beyond 250 MΩ. Therefore, a tightly coupled horizontal cell syncytium, through increased gap junctional conductance, gave rise to damped oscillations of larger amplitude. The junctional conductance had little effect on the spectral range of oscillation, but did increase the power of the oscillation with decreasing junctional resistance (Fig. 8f).

We also simulated a network containing only horizontal cells, interconnected with gap junctions (Fig. 8g). In this network, without reciprocal synapses, we never observed oscillatory behavior over a variety of junctional resistances (Fig. 8h), illustrating the inability of such a network to generate damped oscillations.

## Discussion

In this study, we began by surveying the electrically evoked responses for neurons in the inner two layers of the retina, over a variety of stimulus configurations. Analyses of these data revealed that: (i) the response amplitude of wide-field amacrine cells and gap-junction-coupled horizontal cells were charge insensitive; (ii) some neurons were sensitive to stimulus pulse width, resulting in a change of the most strongly driven cell types with changing pulse width (at a fixed charge of 20 nC); and (iii) damped oscillations, arising from reciprocal excitatory and inhibitory synapses between the cones and horizontal cells, were widespread throughout the retina following a single stimulus pulse. Finally, computational analyses suggested that the oscillatory amplitude can be modulated by gap junctional conductance.

There are three major electrode placement locations for a retinal prosthesis: epiretinal, subretinal and suprachoroidal. The electrodes are placed adjacent to the RGC layer, adjacent to the photoreceptor layer, or in the suprachoroidal space, respectively. By virtue of its close proximity to the RGCs, epiretinal stimulation is able to directly stimulate these neurons while avoiding retinal network activation^45^. The spatiotemporally precise RGC spikes thus elicited could match, or even exceed, the spike timing precision of visually evoked responses in the same neurons^20^. It is not known whether the other placement strategies are able to replicate the neural code of natural vision with such fidelity. We found that a single subretinal pulse generated responses lasting several hundred milliseconds, across multiple layers of the retina. Oscillatory or phasic RGC spiking responses have been observed previously^35,45–47^. Though pharmacological manipulations, these responses were broadly attributed to source(s) presynaptic to the RGCs. Our observations are consistent with these studies, and in particular, we also identified a network substrate responsible for these responses. Given the architectural similarities between the primate and rabbit retina, and more importantly, the simplicity of the network substrate required to create such damped oscillations, we anticipate similar responses to occur also in the human retina following subretinal and suprachoroidal stimulation. Therefore, clinical stimulation protocols should take these long-duration responses into account, when a sequence of well-timed RGC spikes are needed to encode specific visual features.

The effects of stimulus charge on the RGCs have been extensively investigated^35,45,48–50^. Much like the prior RGC findings, we found that stimulus charge variations generally had the same effects on neurons in the inner retina – a positive correlation between response amplitude and charge. The exceptions were two wide-field amacrine cells and the horizontal cells. All three types are spatially extensive, either due to dendritic branching or through high-conductance, gap-junction-mediated syncytium. These anatomical features may have influenced the charge sensitivity of these neurons.

In the retina, visual information is conveyed by several parallel information streams^51^, each represented by different cell types. There has been a long-standing interest in preferential activation of retinal neurons with electrical stimulation. Recent work by Twyford *et al*.^52^ offered the possibility of preferentially recruiting RGC types using high-frequency pulse trains. Analysing a wide variety of retinal neurons, across a range of stimulation configurations, we found here that certain neuronal types in the inner retina could be preferentially activated through pulse-width variations. By increasing the repertoire of preferential stimulation techniques, it may eventually be possible to target specific cell types, across multiple retinal layers, thereby improving the clinical efficacy of retinal prosthesis through better device-to-neuron interfacing.

Based on RGC spike train analyses, it has been suggested that short-duration pulses are effective at eliciting direct RGC responses, and that long-duration pulses are more effective for evoking responses from the interneurons^35,45,49^. We observed a shift in the most strongly driven cell type as the pulse width increased, thus supporting the notion that some neurons may be preferentially targeted with specific choices of pulse width. A similar strategy has been used recently by Weitz and colleagues^53^ to achieve spatially focused excitation with 25-ms pulses, which was postulated to preferentially stimulate bipolar cells.

Two factors influenced our sample size in this study. First, there is great diversity in retinal cell types. Many of these are thought to be rare^28^, limiting the probability of encounter. Second, an inherent challenge of interneuron recordings in the intact retina is difficult access. While sharp microelectrodes permitted intracellular recordings without slicing the retina or delaminating the photoreceptor layer, it tends to be less stable than whole-cell recordings, thus limiting the recording duration. Recent developments in genetically labelled retinal neurons^54^ and the increasing availability of these mouse lines should dramatically improve experimental yield by permitting targeted recordings with fluorescent labels.

The retinal network undergoes progressive remodelling in neurodegenerative diseases^24^. We anticipate the results here to serve as a useful benchmark for electrically evoked responses in a healthy neural network, against which subsequent studies in diseased retinas could be compared. Persistent oscillations have been observed in the degenerate retina. The mechanisms underlying these responses have been the focus of recent studies^55–58^. Due to the presence of reciprocal synaptic connections in the degenerate retina, for instance, between the bipolar cells and the amacrine cells, the electrically evoked oscillations we observed here are likely to also occur in the degenerate retina. Furthermore, similar to previous findings in the rd1 and rd10 mice^55,57,58^, the damped oscillations are enhanced by gap junctions. In future studies, it will be important to conduct similar surveys in the diseased retina, at different degeneration stages, to understand the extent to which disease progression could impact vision restoration through electrical stimulation.

## Methods

### Electrophysiology

Adult New Zealand White rabbits weighing 2.0~3.0 kg, were anesthetized with ketamine (70 mg/kg) + xylazine (10 mg/kg). After enucleating both eyes, the animals were euthanized with an overdose of sodium pentobarbital. Inferior retina with the sclera attached was dissected and kept under darkness in Ames’ Medium supplemented with 1% Penicillin/Streptomycin (Invitrogen). After 1~10 hours, small pieces of the retina were isolated and transferred RGC-side up into an imaging chamber. All experimental procedures were approved and monitored by the University of New South Wales (UNSW) Animal Care and Ethics Committee. All animal-related experimental procedures were performed in accordance with the guidelines and regulations defined in the UNSW Animal Research Ethics Procedure document.

The retinal tissue was perfused with Ames’ Medium at 34~35 °C during electrophysiological recordings. Data were low-pass filtered at 10 kHz and digitized at 50 kHz. We recorded the responses of RGCs and displaced starburst amacrine cells with whole-cell patch clamp. The electrodes were filled with (mM): 120 KMeSO_4_, 10 KCl, 0.008 CaCl_2_, 0.5 EGTA, 1 MgCl_2_, 10 HEPES, 4 ATP-Na_2_, 0.5 GTP-Na_3_ and 0.075 Alexa Fluor 488, pH 7.2. The electrode resistance ranged 3.0~5.5 MΩ with the above solution. A calculated and measured 5 mV liquid junction potential has been applied to all results. We conducted intracellular recording of neurons in the inner nuclear layer (INL) using sharp microelectrodes filled with: 1 M KMeSO_4_, 10 mM KCl, 2 mM EGTA and 4% Neurobiotin-Cl. The electrode resistance ranged from 100~150 MΩ. All chemicals, except Alexa Fluor 488 (Life Technologies) and Neurobiotin-Cl (Vector Laboratories), were supplied by Sigma Aldrich.

### Morphological reconstruction and cell identification

For patch clamp recordings, where cells were filled with Alexa Fluor 488, dye loading was achieved by passive diffusion, followed by epi-fluorescence microscopy. For sharp microelectrode recordings, Neurobiotin was loaded by passing +1 nA square pulses at 2 Hz for ≥120 s, followed by 30 minutes of incubation. The retinas were fixed with 4% paraformaldehyde (Sigma Aldrich) in phosphate buffer, incubated in PBS with 0.5% Triton-X and 1% BSA (Sigma Aldrich), reacted against Streptavidin - Alexa Fluor 488 conjugate (Life Technologies), counter stained with DAPI (Life Technologies), then mounted with Pro-long Gold (Life Technologies). The retinas were imaged with a confocal microscope (Olympus FV1000). Amacrine cells were first broadly categorized by dendritic field diameter^28^, namely: narrow field (<125 µm), medium field (125~400 µm) and wide field (>400 µm). Further subdivisions were made on the basis of dendritic stratification, branching patterns, presence of varicosities and dye coupling. When matching our amacrine cells to previously reported types, we used the naming schemes of MacNeil and colleagues^28,29^. The exceptional cells were: MF1, which was called A18 by Kolb^30^ and a TH-IR cell by Casini *et al*.^31^; MF2, which was called A13 by Kolb and Nelson^32^; and WF2, which was called A17 by Nelson and Kolb^33^ and an indoleamine S-2 cell by Vaney^34^. The bipolar cells were classified into the On or the Off subtypes, based on light responses, then confirmed morphologically by dendritic stratification. We identified the A-type horizontal cells by their light-induced hyperpolarization, morphology and extensive gap-junction coupling. The On-off direction selective RGCs (DSGCs) were identified by their responses to both light onset and offset.

### Pharmacology

The AMPA/kainate, NMDA, mGluR6, GABA_a/c_ and glycinergic receptors were blocked by bath application of (µM): 75 6-cyano-7-nitroquinoxaline-2,3-dione (CNQX), 60 (+)-MK 801 hydrogen maleate (MK-801), 20 L-( + )-2-Amino-4-phosphonobutyric acid (L-AP4), 100 picrotoxin (PTX) and 10 strychnine (STY), respectively. Drugs were purchased from either Tocris Bioscience or Sigma Aldrich.

### Electrical stimulation

The wholemount retinas were stimulated at the photoreceptor side (subretinal stimulation) with a 40-µm diameter platinum electrode in a multielectrode array (MEA; Qwane Biosciences) embedded in the base of the imaging chamber. Sixty evenly spaced electrodes were arranged in a square grid within the MEA. The electrode center-to-center distance was 200 µm. The cells were stimulated with a monopolar return configuration. Eight distant and evenly spaced electrodes connected in parallel at the MEA perimeter served as the stimulus return. These distant return electrodes were always at least 400 µm away from the stimulation working electrode. The electrical stimuli were constant-current, charge-balanced, cathodic-first, rectangular biphasic pulses without inter-phase delay. We repeated all stimulus configurations at least 10 times, with 1 s between presentations.

### Light stimulation

Recordings were made at constant mesopic lighting (~7 cd.sr/m^2^). To study the light evoked responses, we projected a 160 µm diameter circular light spot onto the photoreceptor layer and centered over the soma of the recorded neuron. The light stimulus was white and 4 s in duration, consisting of the sequence On-Off-On-Off, with 1 s for each segment.

### Analyses

All results are in the mean ± SEM format. Statistical tests with p < 0.05 (2-tailed) were considered significant. In the stimulus-time plots (e.g. Fig. 1e), the changes in membrane potential (∆*V*_*m*_), averaged over 10 trials, are denoted by color variations. To show the responses in conventional format, we also overlaid in grey a representative single trial response at the highest stimulus amplitude.

To evaluate the effect of stimulus pulse width, we first expressed the electric pulse as the total charge delivered (pulse amplitude × pulse width). Comparisons were then made between pulse widths at identical charge levels, thereby eliminating the effect of pulse amplitude.

The retina contains both spiking and graded responses. Both types were analyzed by the same method. To characterize the perturbation caused by the stimulus independently of the time evolution of the response, we used the following conceptual treatment. The trials were combined to form an average time-waveform. The time-coordinate of the waveform was collapsed by projecting all the ∆*V*_*m*_(*t*) points onto the common vertical (∆*V*_*m*_) coordinate axis. The assemblage of *V*_*m*_ values was treated as samples drawn from a hypothetical probability density function to yield its standard deviation (s_mean ∆Vm_). The latter is the measure of perturbation. In practice, we repeated the stimulus trial 10 times, to compute the average time-waveform. The pre-stimulus data and the stimulus artifacts were excluded from analysis.

We used multi-tapered spectral analysis^59^ to characterize the frequency domain structure of the evoked responses. The spectra were computed from a 390 ms window using three Slepian data tapers at 10 Hz.

### Computational model

The computational network model was implemented in NEURON^60^, using strategies similar to our previous work^61^. It had a three-dimensional topology containing multi-compartmental neurons with conductance-based description of ion channel dynamics. The network contained cone photoreceptors and horizontal cells. We used the cone photoreceptor model developed in Publio *et al*.^62^. The horizontal cell was based on the model of Aoyama and colleagues^63^, modified into three-dimensional form, to contain dendrites. It reproduced all behaviors of the original model. The network contained 13 × 13 cones and 13 × 13 horizontal cells in two planar grids separated by 15 µm. The dendritic fields of neighboring horizontal cells were spatially overlapping to mimic experimental observations. Every cone and horizontal cell was connected to four neighbors by gap junctions. In practice, a many-to-many connectivity exists for the cones and horizontal cells. For computational efficiency, we used a one-to-one relationship. Each cone and horizontal cell pair was connected by an excitatory feed-forward synapse and an inhibitory feedback synapse.

Gap junctions were modeled as resistors connecting two compartments. Chemical synapses followed the general scheme of Destexhe *et al*.^64^, with a stationary relationship between the presynaptic membrane voltage and the post-synaptic membrane current:

1 $${I}_{syn}={g}_{syn}\times 0.5\,\times \,(\tanh (\frac{{v}_{pre}-{v}_{\lambda }}{{v}_{slope}})+1)$$

where *V*_*pre*_ is the presynaptic voltage and *I*_*syn*_ is the postsynaptic current. The parameters *g*_*syn*_, *v*_*λ*_ and *v*_*slope*_ determine the maximum synaptic conductance, half-activation voltage and activation gradient, respectively. When the cones and horizontal cells were connected, their membrane potentials converged to an equilibrium of approximately −49 and −50 mV, respectively. These values were close to the experimental observations.

The network was stimulated above its center position with an extracellular current pulse in a homogeneous medium. The extracellular potential at each point *v(x, y, z)* was determined by a three-dimensional form of the equation in McIntyre and Grill^65^:

2 $$v(x,y,z)=\frac{{I}_{stim}{\rho }_{ext}}{4\pi \sqrt{{(x-{x}_{stim})}^{2}+{(y-{y}_{stim})}^{2}+{(z-{z}_{stim})}^{2}}}$$

where *I*_*stim*_ and ${\rho }_{ext}$ are the extracellular current amplitude and extracellular resistivity, respectively. The stimulus induced trans-membrane current could then be computed for each segment per McIntyre and Grill^65^. Supplementary Table 1 contains further details of the computational model. The electrically evoked responses in the model were comparable to the experimental results.

### Data availability

The datasets generated during and/or analysed during the current study are available from the corresponding author on reasonable request.

## Electronic supplementary material

Supplementary information
