# Supplementary material for: Survey of electrically evoked responses in the retina - stimulus preferences and oscillation among neurons
Source: Sci Rep. 2017 Oct 23;7:13802. doi: 10.1038/s41598-017-14357-1 (PMC5653866; doi:10.1038/s41598-017-14357-1)
Supplement: Supplementary file 1 — Supplementary information [file 41598_2017_14357_MOESM1_ESM.doc]

**Survey of electrically evoked responses in the retina - stimulus preferences and oscillation among neurons**

**David Tsai, John W Morley, Gregg J Suaning, Nigel H Lovell**

# Supplementary figures


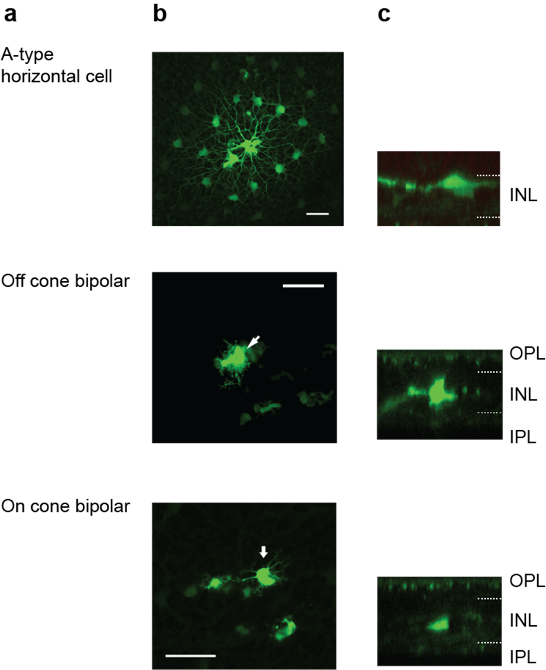


**Supplementary figure 1. Morphology of horizontal cells and cone bipolar cells**

(a) Name of cell type. (b) Representative top view of cell type by confocal z-stack projection. When present, the arrow indicates the somatic location. Scale bar, 30 µm. (c) Somatic location in the inner nuclear layer (INL). OPL and IPL denote outer plexiform layer and inner plexiform layer, respectively.


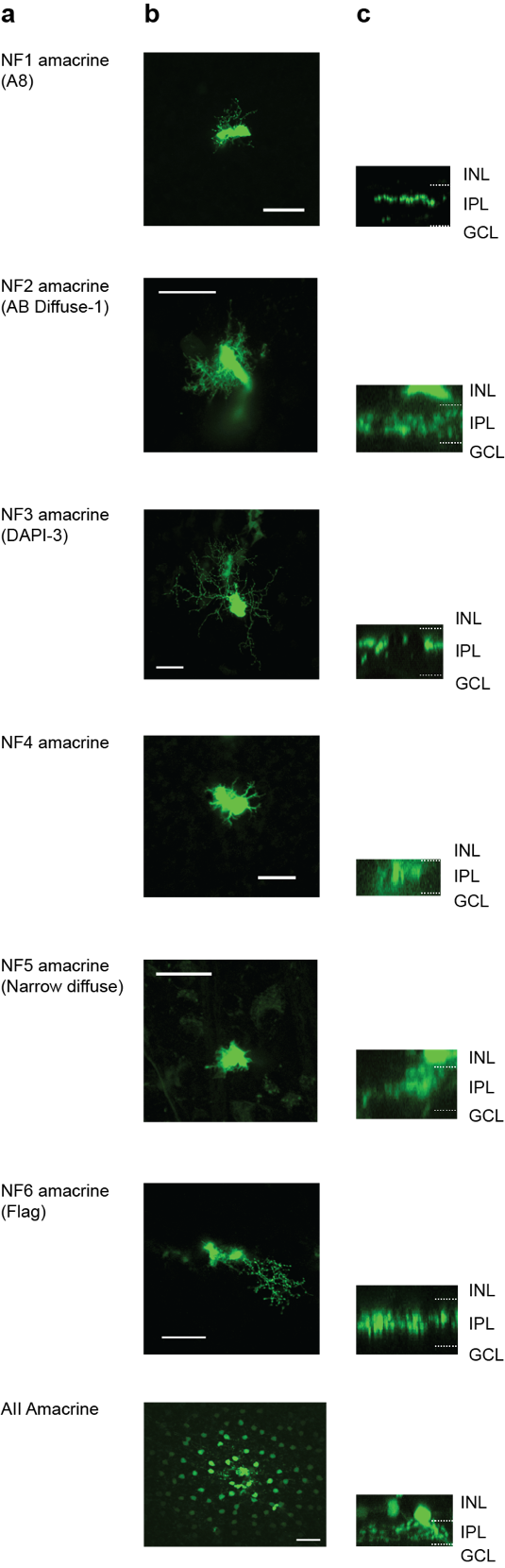


**Supplementary figure 2. Morphology of narrow field (dendritic arbor <125 µm) amacrine cells**

(a) Name of cell type and putative equivalent name in parentheses. (b) Representative top view by confocal z-stack projection. Scale bar, 30 µm. (c) Dendritic stratification in the inner plexiform layer (IPL). INL denotes inner nuclear layer, GCL denotes ganglion cell layer.

**
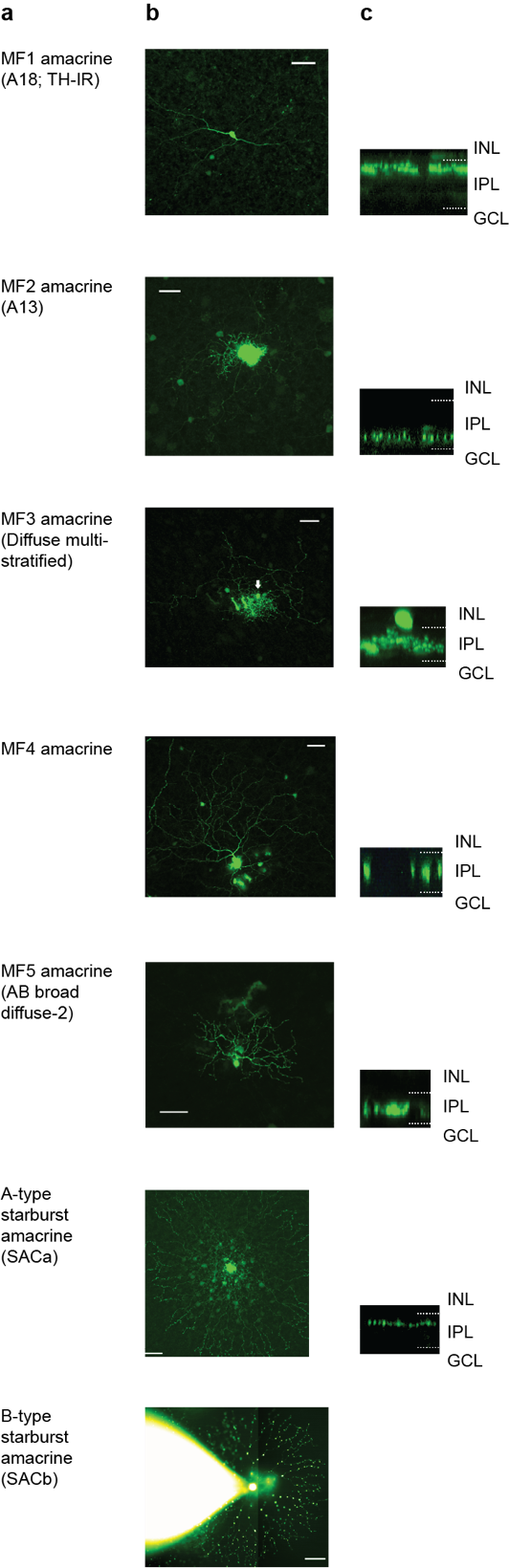
**

**Supplementary figure 3. Morphology of medium field (dendritic arbor 125 – 400 µm) amacrine cells**

See Supplementary fig. 2 legend. Scale bar, 30 µm.


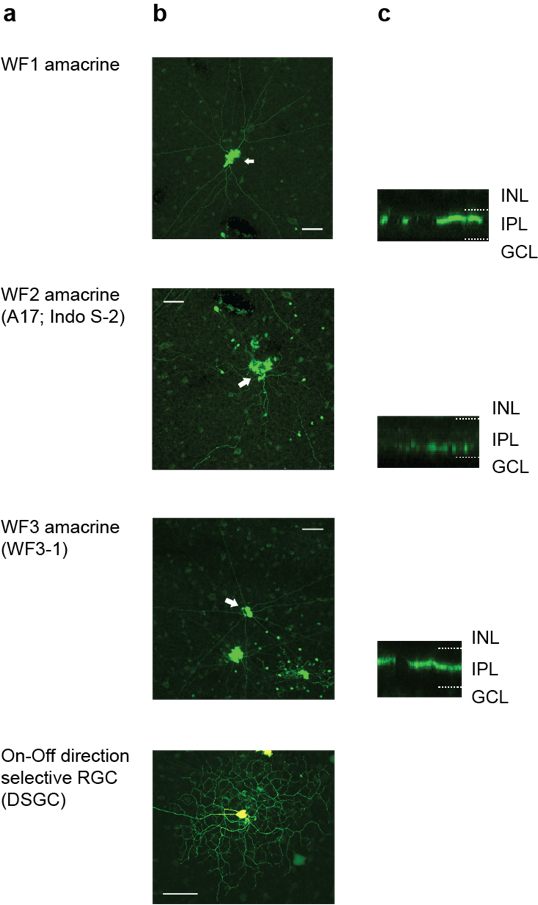


**Supplementary figure 4. Morphology of wide field (dendritic arbor > 400 µm) amacrine cells and On-Off direction selective retinal ganglion cell (DSGC)**

See Supplementary fig. 2 legend. Scale bar, 70 µm.

**Supplementary Table**

**Table 1 Computational model description.**

| **Description** | **Value** |
| --- | --- |
| **Cone photoreceptors**  Per Publio et al. (2009) | |
| Compartments | 1 |
| Geometry | Length = 20 µm, diam = 3.14 µm |
| Ionic currents | I_h_, I_Kv_, I_KCa_, I_Ca_, I_Cl(Ca)_ |
| C_m_ | 32 nC |
| V_init_ | -45 mV |
| **Horizontal cells (Hz)**  Per Aoyama et al. (2000) except for the values in italic | |
| *Compartments* | *9 (1 soma, 8 dendrites)* |
| *Somatic geometry* | *Length = 10 µm, diam = 10 µm* |
| *Dendritic geometry* | *Length = 137 µm, diam = 3 µm* |
| Ionic currents | I_Na_, I_Ca_, I_Kv_, I_A_, I_Ka_, I_L_ |
| R_a_ | 160 MΩ |
| C_m_ | 1 µF/cm^2^ |
| V_init_ | -45 mV |
| **Network topology** | |
| Cone photoreceptors | 169 cells arranged in a 13 x 13 grid |
| Hz cells | 169 cells arranged in a 13 x 13 grid |
| Cone – Hz somatic center separation | 20 µm in z-plan |
| Cone – cone somatic center separation | 50 µm in xy-plan |
| Hz – Hz somatic center separation | 50 µm in xy-plan |
| **Gap junctions** | |
| Cone – cone R_gap_ | 1000 MΩ |
| Cone – cone connection | 4 neighbors, at somatic edge |
| Hz – Hz R_gap_ | 50 ~ 250 MΩ |
| Hz – Hz connection | 4 neighbors, at 0.12*L_dendrite_ point |
| **Excitatory Synapses** | |
| Direction | Cone → Hz |
| Total | 131 (1 per cone – Hz pair) |
| V_λ_ | -47 mV |
| V_slope_ | 5 mV |
| g_max_ | 0.025 µS |
| **Inhibitory Synapses** | |
| Direction | Hz → cone |
| Total | 131 (1 per Hz – cone pair) |
| V_λ_ | -47 mV |
| V_slope_ | 5 mV |
| g_max_ | -0.025 µS |
| **Extracellular Microstimulation** | |
| ρ_ext_ | 110 MΩ |
| Stimulus location | Grid center, 35 µm above cone |
| Pulse width | 0.1 ~ 1.0 ms |
| Pulse amplitude | 0 ~ 300 µA |
| **Intracellular Stimulation**  For a pair of reciprocally connected neurons | |
| Location | Current injection at somatic center |
| Amplitude | 0.4 nA |
| Duration | 0.5 ms |
| **Execution** | |
| Time step | 12.5 µs |
| Temperature | 35 degrees C |
